# Female Specific Association of Low Insulin-Like Growth Factor 1 (IGF1) Levels with Increased Risk of Premature Mortality in Renal Transplant Recipients

**DOI:** 10.3390/jcm9020293

**Published:** 2020-01-21

**Authors:** Frank Klont, Lyanne M. Kieneker, Antonio W. Gomes-Neto, Suzanne P. Stam, Nick H. T. ten Hacken, Ido P. Kema, André P. van Beek, Else van den Berg, Péter Horvatovich, Rainer Bischoff, Stephan J. L. Bakker

**Affiliations:** 1 Department of Analytical Biochemistry, Groningen Research Institute of Pharmacy, University of Groningen, 9713 AV Groningen, The Netherlands; P.L.Horvatovich@rug.nl (P.H.); r.p.h.bischoff@rug.nl (R.B.); 2 Department of Internal Medicine, Division of Nephrology, University Medical Center Groningen, University of Groningen, 9700 RB Groningen, The Netherlands; l.m.kieneker@umcg.nl (L.M.K.); a.w.gomes.neto@umcg.nl (A.W.G.-N.); s.p.stam@umcg.nl (S.P.S.); elsevandenberg@hotmail.com (E.v.d.B.); s.j.l.bakker@umcg.nl (S.J.L.B.); 3 Department of Pulmonary Diseases, University Medical Center Groningen, University of Groningen, 9700 RB Groningen, The Netherlands; n.h.t.ten.hacken@umcg.nl; 4 Department of Laboratory Medicine, University Medical Center Groningen, University of Groningen, 9700 RB Groningen, The Netherlands; i.p.kema@umcg.nl; 5 Department of Endocrinology, University Medical Center Groningen, University of Groningen, 9700 RB Groningen, The Netherlands; a.p.van.beek@umcg.nl

**Keywords:** insulin-like growth factor 1, growth hormone, muscle mass, patient survival, physical activity, renal transplant recipients

## Abstract

Associations between insulin-like growth factor 1 (IGF1) and mortality have been reported to be female specific in mice and in human nonagenarians. Intervention in the growth hormone (GH)-IGF1 axis may particularly benefit patients with high risk of losing muscle mass, including renal transplant recipients (RTR). We investigated whether a potential association of circulating IGF1 with all-cause mortality in stable RTR could be female specific and mediated by variation in muscle mass. To this end, plasma IGF1 levels were measured in 277 female and 343 male RTR by mass spectrometry, and their association with mortality was assessed by Cox regression. During a median follow-up time of 5.4 years, 56 female and 77 male RTR died. In females, IGF1 was inversely associated with risk (hazard ratio (HR) per 1-unit increment in log2-transformed (doubling of) IGF1 levels, 95% confidence interval (CI)) of mortality (0.40, 0.24–0.65; *p* < 0.001), independent of age and the estimated Glomerular filtration rate (eGFR). In equivalent analyses, no significant association was observed for males (0.85, 0.56–1.29; *p* = 0.44), for which it should be noted that in males, age was negatively and strongly associated with IGF1 levels. The association for females remained materially unchanged upon adjustment for potential confounders and was furthermore found to be mediated for 39% by 24 h urinary creatinine excretion. In conclusion, low IGF1 levels associate with an increased risk of all-cause mortality in female RTR, which may link to conditions of low muscle mass that are known to be associated with poor outcomes in transplantation patients. For males, the strongly negative association of age with IGF1 levels may explain why low IGF1 levels were not found to be associated with an increased risk of all-cause mortality.

## 1. Introduction

The peptide hormone insulin-like growth factor 1 (IGF1) is a key mediator of the biochemical/endocrine effects of growth hormone (GH) [1]. Synthesis of IGF1 is regulated by GH and mainly takes place in the liver after which IGF1 is secreted and transported to other tissues, where it acts as an endocrine hormone [2,3]. IGF1 provides a stable, integrated measure of the activity of the somatotropic axis thereby contrasting with GH secretion which is highly variable [3].

Reduced GH and IGF1 signaling extends lifespan in many laboratory models, including worms, yeast, and drosophila [4]. A specific role for IGF1 receptor signaling in mammalian longevity was first established in IGF1 receptor-(haplo) insufficient mice. These mice lived 33% longer than their wildtype littermates, yet this effect was restricted to females [5], which was subsequently confirmed in two follow-up studies in mice [6,7]. A similar link between IGF1 receptor-insufficiency and longevity has been proposed for humans following observations in several studies [8,9,10]. Moreover, IGF1 levels predict better survival in nonagenarians (i.e., people between the age of 90 and 99), and, notably, the corresponding association between IGF1 levels and longevity was found to be female specific [11]. It remains, however, unclear whether circulating levels of IGF1 are also associated with longevity in middle-aged subjects and whether such association is female specific.

Studying the association between IGF1 levels and longevity (survival) in specific patient groups appears to be interesting as well, for example, following the growing interest in ghrelin receptor agonists targeting the GH-IGF1 axis to potentially reverse the anorexia–cachexia syndrome in a variety of conditions, including renal insufficiency [12,13,14,15]. An important mechanism by which stimulation of the GH-IGF1 axis may improve long-term outcome is through stimulation of muscle mass accretion [15,16]. To this regard, a large and growing group of patients that might be worthwhile studying is that of renal transplant recipients (RTR), in which protein–energy wasting is always lurking [17,18,19]. In fact, it has been found that the risk of premature mortality in this population is 6–7 times higher compared to the general population [20], and this risk was particularly high in RTR with low muscle mass, as reflected by low 24 h urinary creatinine excretion [21,22]. Recent studies furthermore suggested that 24 h urinary creatinine excretion may be a noninvasive, easily accessible, inexpensive, and direct measurement of total body muscle mass [19], while this measure is often not included in clinical studies to complement the imaging technique armamentarium which is applied for evaluation of muscle mass in observational and clinical intervention studies [23,24,25].

In this study, we aimed to investigate (1) the nature of the association between circulating levels of IGF1 and mortality in RTR, (2) whether such (potential) association is female specific, and (3) furthermore whether such (potential) association could, in part or as a whole, be mediated by variation in muscle mass, as reflected by 24 h urinary creatinine excretion.

## 2. Experimental Section

### 2.1. Study Population

All RTR (aged ≥ 18 years) that were transplanted at the University Medical Center Groningen (UMCG) and that were one year or longer post-transplantation were approached for participation in this study during outpatient clinic visits between 2008 and 2010, as described previously [26]. The RTR included in this study had no known or apparent systemic diseases (e.g., malignancies, active infections) at inclusion. Written informed consent was obtained from 707 (87%) of the 817 RTR that were initially invited, and plasma IGF1 levels were measured in 620 RTR (76%). For this study, ethical approval has been granted by the UMCG’s review board (METc 2008/186), and the study adheres to the Declaration of Helsinki. The study is registered as ‘TransplantLines Food and Nutrition Biobank and Cohort Study (TxL-FN)’ at ClinicalTrials.gov (NCT identifier ‘NCT02811835′).

### 2.2. Data and Sample Collection

Measurement of clinical parameters has been described in detail previously [26]. Physical activity was assessed with the Short QUestionnaire to ASsess Health-enhancing physical activity (SQUASH) as developed and validated by the Dutch National Institute of Public Health and Environment to assess daily life physical activity in the Dutch adult population [27]. Information on medical history and medication use was obtained from patient records. Diabetes was defined as the use of antidiabetic medication or fasting plasma glucose of at least 7.0 mmol/L. Twenty-four h urine was collected (per strict protocol) a day before the outpatient clinic visits while blood was drawn in the morning on the day of the outpatient clinic visit, yet after completion of the 24 h urine collection.

### 2.3. Laboratory Procedures

Blood and urine markers were measured by routine laboratory procedures with the exception of serum creatinine which was assessed using a modified version of the Jaffé method (MEGA AU 510; Merck Diagnostica, Darmstadt, Germany), and the urine total protein concentration which was obtained using the Biuret reaction (MEGA AU 510; Merck Diagnostica). Renal function was estimated with the 2012 Chronic Kidney Disease Epidemiology (CKD-EPI) Collaboration equation using both serum creatinine and cystatin C [28]. IGF1 was assessed in plasma samples (which had not undergone any previous freeze–thaw cycle) using a semi-automated mass spectrometric IGF1 assay [29] which was validated according to FDA guidelines [30]. The samples were analyzed in 13 analytical runs containing up to 81 clinical samples per run, as well as nine calibration samples and duplicate quality control (QC) samples at three concentrations (i.e., low, midrange, and high IGF1 levels). All runs met the acceptance criteria stipulated in the FDA guidelines thereby featuring 75% (though at least six) of the calibration samples with back-calculated levels within 15% (or 20% for the lowest level calibration sample) of their expected value, and at least 67% of the QC samples (though at least one replicate per QC level) yielding IGF1 levels within 15% of their respective nominal value (see Figures S1 and S2 in the Supplementary Material).

### 2.4. Outcome Ascertainment

All-cause mortality was the primary outcome of this study and was recorded until the end of September 2015. Up-to-date information on patient status was obtained on the basis of a continuous surveillance system of the outpatient program. In case the status of a patient was unknown, general practitioners or referring nephrologists were contacted. There was no loss to follow-up for the outcome. Specific causes of mortality were secondary outcomes of this study. This information was obtained by linking patient numbers to the database of the Dutch Central Bureau of Statistics (CBS) to retrieve causes of mortality reported by physicians. Infectious mortality was defined as mortality from infectious causes [31]. Cardiovascular mortality was defined as mortality caused by cardiovascular pathology, coded by ICD-10 codes I10-I52 [32]. Mortality due to malignancies was defined as mortality caused by malignant diseases. Miscellaneous causes of mortality were defined as other causes of death, not included in mortality from infectious causes, cardiovascular mortality, or mortality due to malignancies.

### 2.5. Statistical Analyses

Data analyses were performed using IBM SPSS Statistics for Windows (version 23.0.0.0; IBM Corp., Armonk, NY, USA) and STATA/SE (version 15.1; StataCorp, College Station, TX, USA). All p-values are two-tailed, and a p-value lower than 0.050 was considered statistically significant. Baseline characteristics are presented according to tertiles of plasma IGF1 levels for female and male RTR. Continuous data are presented as mean with SD for normally distributed variables and as median with interquartile range (IQR) for variables with skewed distributions, whereas categorical variables are presented as percentages. Differences in baseline characteristics across the tertiles were tested by one-way ANOVA, Kruskal–Wallis test, and linear-by-linear association χ^2^ test for normally distributed continuous, skewed continuous, and categorical variables, respectively. Multivariable linear regression was performed to assess associations between patients’ characteristics and plasma IGF1 levels in female and male RTR. Models were included for analyses that were adjusted for age alone, for age and estimated glomerular filtration rate (eGFR), and for multiple variables selected following (automatic) stepwise backward elimination. The prospective associations of plasma IGF1 levels with all-cause mortality, as primary endpoint, and with cause-specific mortality, as secondary endpoint, were assessed by Cox proportional hazards regression. In order to verify the existence of effect modification by sex for the primary endpoint, we performed Cox regression analyses for the association of plasma IGF1 levels with all-cause mortality in which female and male RTR were grouped together, with additional inclusion of an interaction term of plasma IGF1 and sex in the Cox regression model. Hazard ratios (HR) and 95% confidence intervals (CIs) were calculated per 1 unit increment in log2-transformed IGF1 levels. Thereafter, we proceeded with sex-stratified prospective analyses for all-cause mortality as a primary endpoint. In addition to crude analyses, we performed analyses with adjustment for age and eGFR with and without additional physiological, lifestyle, routine clinical chemistry, transplantation, medication, and comorbidity related variables. Subsequently, we performed mediation analyses using the method as described by Preacher and Hayes, which allowed for testing the significance and magnitude of (potential) mediation [33]. In these analyses, mediation was assessed by computing bias-corrected confidence intervals upon running 2000 bootstrap samples. The proportion of mediation was obtained by dividing the indirect effect coefficient by the total effect coefficient, which were adjusted for age and eGFR. Mediation analyses were performed using IGF1 as a potential risk factor and 24 h urinary creatinine excretion, a marker of muscle mass, as potential mediator while also vice versa, because the observational nature of our study does not allow for drawing conclusions on cause–effect relationships. At last, we performed Cox-regression analyses for the association of plasma IGF1 levels with cause-specific mortality as secondary endpoints. Due to lower numbers of events, the exploratory nature of these analyses, and the generally accepted rule of thumb that allows for one variable to be included for each 7–10 events in Cox regression models [34], we performed sex-stratified crude analyses Cox regression analyses and sex-stratified age- and eGFR-adjusted analyses for the separate causes of mortality.

## 3. Results

### 3.1. RTR Characteristics

**Figure 1:**
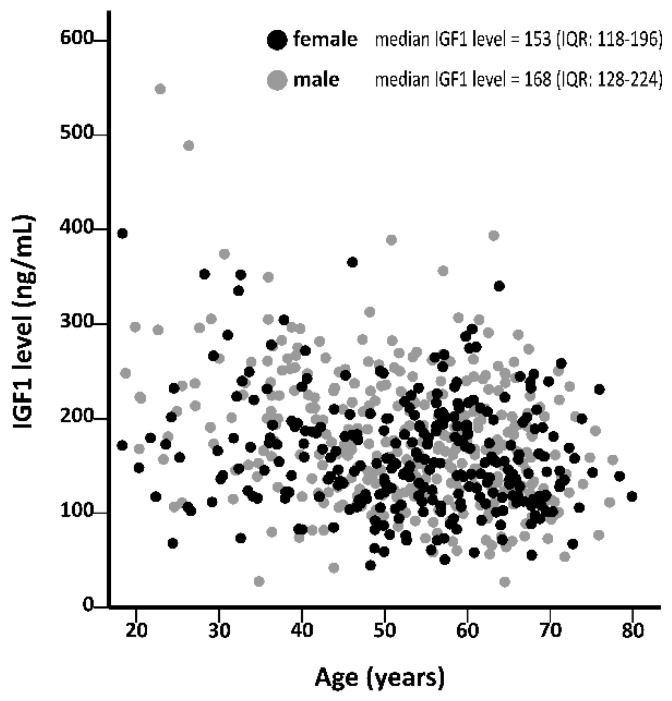
Association between insulin-like growth factor 1 (IGF1) levels and age for female and male renal transplant recipients (RTR).

Baseline characteristics according to tertiles of plasma IGF1 levels for female and male RTR are shown in Table 1. At baseline, median IGF1 levels were 153 ng/mL (IQR: 118–196) in female and 168 ng/mL (IQR: 128–224) in male RTR (see Figure 1).

Female RTR who had higher IGF1 levels were more likely to have a larger waist circumference and a higher 24 h urinary creatinine excretion. In turn, the prevalence of diabetes mellitus as primary renal disease, the use of insulin therapy, the cumulative prednisolone dose, and the time between transplantation and baseline measurements were lower for these subjects.

For male RTR with higher levels of IGF1, subjects were more likely to be younger, to have a higher body weight and SQUASH score, and to have a lower waist circumference, prevalence of diabetes mellitus as primary renal disease, and cumulative prednisolone dose. Male RTR in the highest tertile of IGF1 levels were furthermore more likely to have received a graft from a living donor, to have undergone dialysis before transplantation, to have a shorter time between transplantation and baseline measurements, to use calcineurin inhibitors, whereas these subjects were less likely to use coumarin derivatives. Lastly, levels of serum creatinine, plasma albumin, plasma triglycerides, and 24 h urinary creatinine excretion were more likely to be higher whereas plasma aspartate transaminase (AST), gamma-glutamyltransferase (GGT), and high sensitivity C-reactive protein (hs-CRP) were more likely to be lower for these subjects.

### 3.2. Association of Plasma IGF1 with Selected Variables in RTR

Associations between plasma IGF1 levels and variables of interest adjusted for age alone, for age and eGFR, and for multiple variables which were selected following stepwise backward elimination are shown in Table 2.

For female RTR, the analyses adjusted for age featured positive and significant associations between plasma IGF1 and body weight, 24 h urinary creatinine excretion, and calcineurin inhibitor use. Significant inverse associations with plasma IGF1, independent of age, were observed for the prevalence of diabetes mellitus as primary renal disease, the time between transplantation and baseline measurements, the cumulative prednisolone dose, and GGT. After further adjustment for eGFR, the magnitude, direction, and significance of all associations generally remained the same. The final stepwise backward model featured an adjusted R^2^ of 0.14 and revealed significant positive associations between plasma IGF1 and both 24 h urinary creatinine excretion and calcineurin inhibitor use, but also significant inverse associations with the prevalence of diabetes mellitus as primary renal disease, GGT, HDL cholesterol, and hs-CRP.

For male RTR, analyses adjusted for age showed significant positive associations between plasma IGF1 and both albumin and calcineurin inhibitor use. Significant inverse associations were observed between plasma IGF1 and eGFR, the prevalence of diabetes mellitus as primary renal disease, the cumulative prednisolone dose, AST, and GGT, and the time between transplantation and baseline measurements. Further adjustment for eGFR did not lead to major changes in the magnitude, direction, or significance of these associations. Lastly, an adjusted R^2^ of 0.28 was obtained for the final stepwise backward model which featured significant positive associations between plasma IGF1 and both albumin and calcineurin inhibitor use. Significant inverse associations were furthermore revealed between plasma IGF1 and age, eGFR, the prevalence of diabetes mellitus as primary renal disease, and GGT.

### 3.3. Association of Plasma IGF1 with All-Cause Mortality in RTR

**Figure 2:**
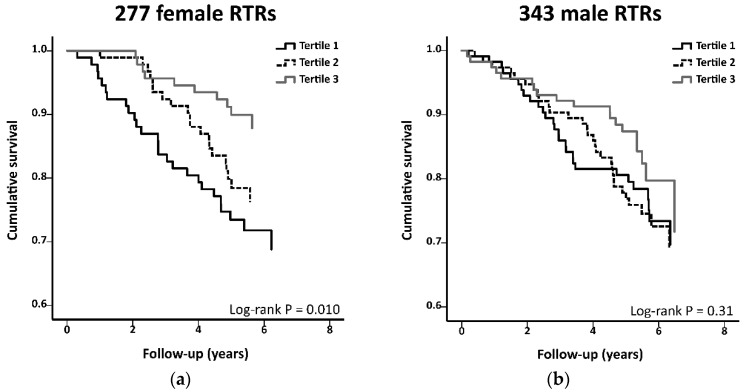
Kaplan–Meier curves for all-cause mortality according to tertiles of plasma insulin-like growth factor 1 (IGF1) in (**a**) female and (**b**) male renal transplant recipients (RTR). For female RTR, IGF1 levels of the tertiles 1, 2, and 3 are below 131 ng/mL, range between 131 and 181 ng/mL, and are above 181 ng/mL, respectively. For male RTR, IGF1 levels of the tertiles 1, 2, and 3 are below 141 ng/mL, range between 141 and 202 ng/mL, and are above 202 ng/mL, respectively.

Median follow-up was 5.4 years (IQR: 4.8–6.0 years) for female and 5.4 years (IQR: 4.8–6.3 years) for male RTR. During this prospective follow-up, 56 female and 77 male RTR died. We first investigated whether the association of plasma IGF1 levels with all-cause mortality was modified by sex. In these analyses, with data of female and male RTR combined, we found that higher plasma IGF1 levels were associated with a significantly decreased risk (HR per log2 increment of plasma IGF1, 95% CI) of all-cause mortality (0.61, 0.47–0.80; *p* < 0.001). Furthermore, inclusion of a product-term of (log2-transformed plasma) IGF1 levels and sex in the basic multivariable model (i.e., with adjustment for age and eGFR) revealed the existence of significant effect modification by sex (*p* for interaction = 0.02). After finding this significant interaction by sex, we proceeded with sex-stratified analyses of the association of (log2-transformed) plasma IGF1 levels with all-cause mortality. For female RTR, the crude analyses showed that higher plasma IGF1 levels were associated with a significantly decreased risk of all-cause mortality (0.42, 0.26–0.66; *p* < 0.001; see Figure 2 and Table 3), while a nonsignificant trend towards a decreased risk was observed for male RTR (0.74, 0.52–1.04; *p* = 0.09; see Table 3 and Figure 2).

In the model with adjustment for age and eGFR, the significant inverse association of IGF1 with all-cause mortality remained in female RTR (0.40, 0.24–0.65; *p* < 0.001) and the association in male RTR remained insignificant (0.85, 0.56–1.29; *p* = 0.44). Further adjustment for potential confounders, which was assessed based on seven different multivariable models, did not substantially affect the associations between plasma IGF1 and mortality for both female and male subjects (see Table 3). Lastly, mediation analysis (according to the procedures of Preacher and Hayes [33]) was carried out for the female subjects and revealed 24 h urinary creatinine excretion as significant mediator (*p*-value for indirect effect < 0.05) accounting for 39% on the association between plasma IGF1 and all-cause mortality (see Table 4). Since the observational nature of our study does not allow for drawing conclusions regarding cause–effect relationships, we also performed alternative mediation analyses with 24 h urinary creatinine excretion as potential risk factors and plasma IGF1 levels as potential mediators. In these analyses, we found that plasma IGF1 levels as significant mediators (*p*-value for indirect effect < 0.05) accounted for 9% on the association between 24 h urinary creatinine excretion and all-cause mortality (see Supplemental Table S5).

### 3.4. Association of Plasma IGF1 with Cause-Specific Mortality in RTR

Next, we performed sex-stratified analyses of the association of log2-transformed plasma IGF1 levels with mortality from specific causes of death, namely death from infectious diseases, cardiovascular mortality, death from malignancies, and other, miscellaneous causes of death. In females, we found that higher plasma IGF1 levels were strongly associated with a significantly decreased risk of infectious disease-related mortality (0.17, 0.07–0.38; *p* < 0.001; see Supplemental Table S1, Model 1). In females, we also found a borderline significant association of higher plasma IGF1 levels with cardiovascular mortality (0.43, 0.18–1.00; *p* = 0.05; see Supplemental Table S2, Model 1), but neither a significant association with cancer-related mortality (1.50, 0.45–4.93; *p* = 0.51; see Supplemental Table S3, Model 1), nor with mortality from miscellaneous causes (0.43, 0.10–1.78; *p* = 0.24; see Supplemental Table S4, Model 1). In males, no significant associations with cause-specific mortality were encountered (see respective Supplemental Tables S1–S4).

## 4. Discussion

This study showed that low plasma IGF1 levels were independently associated with an increased risk of all-cause mortality in female RTR. Such association was less pronounced and insignificant in male RTR, which should be seen in the context of IGF1 levels being negatively and strongly associated with age in males, which may explain why low plasma IGF1 levels were not associated with mortality in males. Adjustment for potential confounders did not alter the association observed in women, and 39% of this association was found to be mediated by 24 h urinary creatinine excretion, a marker of muscle mass. In alternative analyses, we found that 9% of the association of urinary creatinine excretion with mortality in women was mediated by plasma IGF1 levels. In secondary analyses, in which the association of plasma IGF1 with cause-specific mortality was assessed, we found a particularly strong association of low plasma IGF1 levels with increased risk of mortality due to infectious causes in females.

To our knowledge, this is the first study that investigated the association between IGF1 and long-term outcomes in RTR, hence we were limited in comparing our study with existing literature. Studies addressing associations between IGF1 and outcomes in other clinical settings are available, yet such studies are scarce and generally do not assess female and male subjects separately. When attempting to compare our results to studies on IGF1 in which both sexes were analyzed separately, we found inconsistent evidence. For example, in a cross-sectional study of 5388 US adults, the magnitude of the (positive) association between high IGF1 levels and the risk of chronic kidney disease was found to be stronger for males than for females [36]. In addition, a study of 183 healthy nonagenarians (i.e., people between the age of 90 and 99) reported a significant association between low IGF1 levels and longer survival in female subjects which was not observed for males [11]. Recently, a prospective population-based study on 1618 elderly adults reported that men featured greater decreases in IGF1 and its most important binding protein (i.e., IGF binding protein 3) with age as compared to females [37]. A recently described cross-sectional study on 200 elderly subjects furthermore reported a (negative) association between IGF1 levels and co-existent frailty and low muscle mass in female subjects whereas such association was not found for male subjects [38]. The difference between females and males as we observed in our study therefore links to previous data but also connects to why gender-specific reference ranges for IGF1 are being employed in routine clinical practice [39,40,41]. It should, however, be noted that all these results were obtained using different analytical methods, and it is known that different methods may yield different analyte levels, particularly in the case of IGF1 [42,43]. Moreover, IGF1 predominantly circulates being bound to IGF binding proteins [44], and the efficiency of dissociating such complexes may vary between (immuno)assays from different vendors and thereby lead to biased, or at least to incomparable results [45].

With respect to the observed association between IGF1 and mortality in female RTR, several other findings which were put forward in our study should be taken into consideration. Firstly, the identification of 24 h urinary creatinine excretion as a strong mediator in this association represents an interesting finding of our study. The fact that 24 h urinary creatinine is a widely available and accepted marker reflecting muscle mass [46,47,48] and the recognition of IGF1 as a growth hormone involved in muscle growth [49,50] support the biological plausibility of a link between IGF1 and physical fitness. Low physical activity is, in fact, known to be a risk factor for morbidity and mortality in RTR [51,52,53], hence further studies on IGF1 in this context are warranted. Secondly, the significant association between IGF1 and the use of calcineurin inhibitors should be viewed in this context as well. The target of these drugs, calcineurin, has been described as a regulator of muscle mass, although it should be noted that much is still unknown about the underlying mechanisms [54,55,56]. Thirdly, the observed strongly significant association of higher plasma IGF1 levels with lower risk for infectious disease-related mortality may be interesting as well in this regard. At last, it should be noted that evidence for a potential link between IGF1 and physical fitness is currently still circumstantial and that further research is needed to verify and explore our findings.

Important limitations of this study include the facts that it represents a single-center study and that it addresses a population consisting mainly of Caucasian participants. It is unknown whether our findings can be extrapolated to other populations, and repeating this study in other populations is therefore desirable. Moreover, there may be untested or residual confounding relevant for the observed association, as is often true for observational studies. Moreover, laboratory markers were analyzed only once at baseline, hence corresponding changes over time could not be addressed in the present study. With respect to the IGF1 measurements, it should be noted that measurements were carried out using biobanked samples which had been stored for several years at −80 °C. Sample stability parameters (e.g., freeze-thaw stability, benchtop stability) were addressed during validation of our IGF1 method thereby following the US Food and Drug Administration (FDA) guidelines on bioanalytical method validation [30]. Nonetheless, storage conditions comparable to those applying to the long-term stored plasma samples could not possibly be addressed during validation, as is often the case when targeting biobanked samples. We could, however, monitor the extent of IGF1 oxidation which represents a prominent feature of our mass spectrometric IGF1 assay [29] considering that protein oxidation is a (unwanted) chemical modification occurring during storage of proteins [57]; yet, no abnormalities in IGF1 oxidation were observed. In order to reduce the (potential) impact of corresponding pre-analytical variability on the quality of our data, we only included samples which had not undergone any previous freeze–thaw cycle and we verified that the samples had not been exposed to deviating storage conditions, for example caused by power outages or freezer malfunctions.

Strengths of this study are its prospective design, the relatively large cohort of well-characterized, stable RTR, the complete follow-up for all-cause mortality, the availability of detailed data on potential confounders, and the use of a mass spectrometric IGF1 assay which allowed for highly selective IGF1 quantification.

In conclusion, low plasma IGF1 levels were found to be associated with an increased risk of all-cause mortality in female RTR, and this association was not found (to be significant) for male RTR. The association in females was mediated for a substantial proportion by 24 h urinary creatinine excretion which hints at a possible link with conditions of low muscle mass (e.g., poor physical fitness, poor nutritional state). Secondary analyses pointed towards a particularly strong association of low plasma IGF1 levels with mortality from infectious causes. Further research is, however, needed to explore the existence and/or relevance of such a link, and also to investigate whether IGF1 can be useful as a (predictive) marker of mortality in female RTR possibly by reflecting physical fitness in this population.

## Figures and Tables

**Table 1:** Baseline characteristics according to tertiles of plasma IGF1 levels in female and male RTR ^1^.

	Tertiles of Plasma IGF1 Levels for 277 Female RTR				Tertiles of Plasma IGF1 Levels for 343 Male RTR			
Variable	<131 ng/mL	131–181 ng/mL	>181 ng/mL	*p*-Value for Trend ^2^	<141 ng/mL	141–202 ng/mL	>202 ng/mL	*p*-Value for Trend ^2^
Age, y	56 (48–64)	54 (44–63)	54 (41–60)	0.42	59 (48–65)	55 (46–61)	49 (38–61)	<0.001
BMI, kg/m^2^	25 (22–30)	26 (23–30)	27 (23–30)	0.31	25 (23–28)	26 (24–30)	26 (23–28)	0.07
Body weight, kg	67 (61–84)	73 (65–81)	74 (65–86)	0.20	80 (73–90)	86 (76–97)	84 (74–93)	0.03
Body length, cm	167 (161–171)	167 (162–173)	168 (163–171)	0.49	179 (174–183)	180 (174–184)	180 (174–185)	0.29
Waist circumference, cm	90 (78–104)	95 (87–106)	95 (88–105)	0.04	100 (91–108)	104 (94–112)	99 (89–107)	0.01
Blood pressure, systolic, mmHg	133 (121–147)	131 (120–142)	132 (124–145)	0.47	138 (127–151)	135 (124–145)	136 (125–145)	0.46
Blood pressure, diastolic, mmHg	80 ± 11	80 ± 12	82 ± 10	0.15	83 ± 10	84 ± 12	84 ± 10	0.27
Lifestyle:								
Smoking status, current, %	11.6	8.7	9.0	0.56	9.8	17.4	16.4	0.19
Alcohol consumption, yes, %	87.2	83.3	79.3	0.18	88.0	91.3	92.5	0.28
SQUASH score, ×1000	4.9 (2.6–7.6)	4.3 (1.7–7.1)	5.0 (2.0–6.7)	0.60	5.2 (1.8–7.3)	6.4 (3.0–10.7)	5.4 (2.5–9.6)	0.02
Primary renal disease:								
Primary glomerulosclerosis, %	23.9	20.4	27.2	0.60	28.9	39.5	32.2	0.61
Glomerulonephritis, %	10.9	8.6	4.3	0.10	9.6	4.4	8.7	0.79
Polycystic kidney disease, %	19.6	24.7	27.2	0.23	14.0	19.3	18.3	0.40
Renal hypoplasia/dysplasia, %	4.3	5.4	2.2	0.45	2.6	3.5	5.2	0.31
Diabetes mellitus, %	8.7	5.4	0.0	0.005	11.4	4.4	1.7	0.002
Other primary renal diseases, %	32.6	35.5	39.1	0.36	32.5	28.9	33.9	0.81
Kidney and transplantation related variables:								
eGFR, mL/min per 1.73 m²	42 (27–57)	45 (32–57)	39 (25–51)	0.31	45 (32–61)	43 (29–55)	41 (31–55)	0.19
Serum creatinine, µmol/L	110 (87–148)	107 (87–140)	118 (96–162)	0.23	128 (102–167)	137 (115–171)	148 (116–175)	0.04
Living donor, %	28.3	40.2	39.1	0.13	26.3	31.9	42.6	0.009
Graft rejection, %	21.7	22.6	18.5	0.59	37.7	32.5	27.0	0.08
Dialysis before transplantation, %	87.0	75.3	78.3	0.14	91.2	89.5	79.1	0.007
Time between transplantation and baseline visit, y	7.5 (3.4–12.5)	5.0 (1.9–14.6)	3.7 (1.1–7.9)	0.003	7.5 (4.2–13.7)	7.0 (2.9–13.9)	2.6 (1.0–7.5)	<0.001
Blood markers:								
ALT, U/L	18 (13–25)	18 (13–22)	18 (13–23)	0.81	21 (16–29)	20 (16–27)	19 (14–27)	0.32
AST, U/L	23 (18–29)	22 (18–26)	20 (18–25)	0.08	24 (20–31)	22 (19–26)	21 (17–25)	<0.001
GGT, U/L	29 (19–49)	25 (16–37)	25 (18–34)	0.09	32 (21–48)	27 (19–43)	24 (18–33)	0.004
Albumin, g/L	42 (40–45)	42 (41–45)	43 (42–45)	0.10	42 (41–45)	43 (41–44)	44 (42–45)	0.003
Glucose, mmol/L	5.0 (4.6–6.2)	5.2 (4.7–5.9)	5.2 (4.7–5.6)	0.66	5.4 (4.9–6.2)	5.3 (4.9–6.2)	5.3 (5.0–5.9)	0.86
HbA1c, %	5.7 (5.4–6.0)	5.9 (5.5–6.3)	5.9 (5.5–6.3)	0.39	5.8 (5.5–6.2)	5.8 (5.5–6.3)	5.8 (5.5–6.2)	0.68
Triglycerides, mmol/L	1.7 (1.2–2.6)	1.7 (1.3–2.1)	1.7 (1.3–2.3)	0.97	1.6 (1.1–2.2)	1.7 (1.2–2.9)	1.8 (1.4–2.3)	0.03
Total cholesterol, mmol/L	5.5 (4.6–6.4)	5.1 (4.4–6.1)	5.2 (4.6–5.9)	0.32	5.1 (4.3–5.9)	4.8 (4.2–5.6)	4.8 (4.3–5.6)	0.29
HDL cholesterol, mmol/L	1.6 (1.1–1.9)	1.4 (1.2–1.8)	1.4 (1.2–1.8)	0.22	1.3 (1.1–1.6)	1.2 (0.9–1.4)	1.2 (1.0–1.4)	0.05
LDL cholesterol, mmol/L	3.0 (2.3–3.7)	3.0 (2.2–3.9)	3.0 (2.5–3.5)	0.94	2.9 (2.3–3.7)	2.8 (2.3–3.5)	2.8 (2.3–3.5)	0.43
hs-CRP, mg/L	1.8 (0.9–5.2)	2.0 (0.9–5.4)	1.7 (0.8–3.0)	0.23	1.8 (0.8–5.5)	1.9 (0.8–5.1)	1.3 (0.5–3.4)	0.03
Follicle-stimulating hormone, U/L	52 (7–90)	51 (5–81)	47 (5–78)	0.87	5.2 (3.0–11.0)	5.7 (3.8–10.7)	4.9 (2.9–8.3)	0.20
Follicle-stimulating hormone ≥ 34 U/L, yes, % ^3^	64.0	65.2	63.6	0.95	3.8	5.8	1.8	0.43
Luteinizing hormone, U/L	32 (8–55)	28 (6–56)	32 (8–58)	0.95	5.0 (3.4–8.3)	5.1 (3.6–8.5)	5.1 (3.4–6.9)	0.52
Urine markers:								
Urinary creatinine excretion, mmol/24 h	8.6 (7.3–10.1)	9.6 (8.0–11.3)	10.1 (9.0–11.3)	<0.001	12.3 (10.3–14.5)	13.1 (11.0–15.4)	13.6 (11.4–15.7)	0.008
Urine total protein, g/24 h	0.14 (0.02–0.47)	0.15 (0.02–0.29)	0.15 (0.02–0.29)	0.67	0.24 (0.02–0.52)	0.25 (0.02–0.59)	0.21 (0.02–0.34)	0.29
Medication use:								
Proliferation inhibitors, yes, %	81.5	84.9	83.7	0.69	79.8	86.8	80.0	0.98
Coumarin derivatives, yes, %	14.1	9.7	7.6	0.15	16.7	11.4	7.8	0.04
Calcineurin inhibitors, yes, %	54.3	51.6	66.3	0.10	48.2	55.3	80.0	<0.001
Sirolimus, yes, %	1.2	4.5	0.0	0.55	4.6	0.9	1.0	0.07
Antihypertensive drugs, yes, %	87.0	81.7	89.1	0.67	88.6	92.1	91.3	0.48
Statins, yes, %	48.9	55.9	51.1	0.57	53.1	60.5	43.5	0.14
Diabetes, yes, %	28.3	23.7	26.1	0.74	27.2	22.8	20.0	0.20
Antidiabetics, yes, %	21.7	15.1	13.0	0.11	17.5	15.8	12.2	0.26
Metformin, yes, %	3.3	4.3	4.3	0.71	7.9	2.6	3.5	0.11
Insulin therapy, yes, %	17.4	10.8	7.6	0.04	10.5	7.9	6.1	0.22
Prednisolone, yes, %	97.8	98.9	98.9	0.54	100.0	97.4	100.0	1.00
Prednisolone, cumulative dose, g ^4^	23 (10–37)	19 (6–40)	11 (4–25)	0.01	23 (14–42)	23 (10–46)	10 (4–28)	<0.001

^1^ Continuous variables are reported as ‘mean ± standard deviation’ when normally distributed (Shapiro–Wilk test *p* ≥ 0.05) or ‘median (interquartile range)’ when not normally distributed (Shapiro–Wilk test *p* < 0.05), and categorical variables are reported as percentage. ^2^ Determined by linear-by-linear association χ^2^ test (categorical variables), Kruskal–Wallis test (continuous variables, not normally distributed), or one-way ANOVA (continuous variables, normally distributed). ^3^ A follicle-stimulating hormone cut-off level of ≥ 34 U/L was used to derive a surrogate marker of post-menopause [35]. Abbreviations: ALT: Alanine transaminase; AST: Aspartate transaminase; BMI: Body mass index; eGFR: Estimated glomerular filtration rate; GGT: Gamma-glutamyltransferase; HbA1c: Glycated hemoglobin; HDL: High-density lipoprotein; hs-CRP: High sensitivity C-reactive protein; LDL: Low-density lipoprotein; SQUASH: Short QUestionnaire to ASsess Health-enhancing physical activity [27]. ^4^ The cumulative dose of prednisolone was calculated as the sum of the maintenance dose of prednisolone until inclusion and the dose of prednisolone or methylprednisolone required for treatment of acute rejection (a conversion factor of 1.25 was used to convert methylprednisolone dose to its prednisolone dose equivalent).

**Table 2:** Multivariable linear regression analysis with plasma IGF1 as the dependent variable in female and male RTR ^1^.

	Female RTR (N = 277)						Male RTR (N = 343)					
	Age Adjusted		Age and eGFR Adjusted		Backward (adj. *R*^2^ = 0.14)		Age Adjusted		Age and eGFR Adjusted		Backward (adj. *R*^2^ = 0.28)	
Variable	Stand. β	*p*-Value	Stand. β	*p*-Value	Stand. β	*p*-Value	Stand. β	*p*-Value	Stand. β	*p*-Value	Stand. β	*p*-Value
Age, y	−0.16	0.007	−0.17	0.006			−0.32	<0.001	−0.35	<0.001	−0.27	<0.001
eGFR, mL/min per 1.73 m²	−0.05	0.37	−0.05	0.37			−0.18	0.001	−0.18	0.001	−0.19	0.001
Body weight, kg	0.12	0.05	0.11	0.06			0.01	0.87	−0.01	0.88		
Body length, cm	0.09	0.14	0.09	0.14			0.00	0.97	−0.02	0.74		
SQUASH score	−0.05	0.43	−0.04	0.49	−0.11	0.09	0.02	0.64	0.05	0.38		
Diabetes mellitus, yes vs. no	−0.13	0.04	−0.13	0.03	−0.13	0.04	−0.12	0.02	−0.14	0.008	−0.13	0.009
Living donor, yes vs. no	0.02	0.71	0.03	0.63			0.02	0.66	0.04	0.44		
Graft rejection, yes vs. no	−0.05	0.37	−0.07	0.28			−0.10	0.06	−0.10	0.05	−0.09	0.06
Dialysis before transplantation, yes vs. no	0.07	0.28	0.07	0.23			0.05	0.39	0.06	0.26		
Time between transplantation and baseline visit, y	−0.14	0.02	−0.14	0.02			−0.15	0.003	−0.15	0.004		
AST, U/L	−0.09	0.16	−0.09	0.16			−0.21	<0.001	−0.15	0.005	−0.10	0.06
GGT, U/L	−0.12	0.04	−0.13	0.03	−0.14	0.02	−0.22	<0.001	−0.20	<0.001	−0.18	0.001
Albumin, g/L	0.09	0.15	0.10	0.11			0.12	0.03	0.17	0.003	0.15	0.01
Triglycerides, mmol/L	−0.06	0.36	−0.07	0.23	−0.11	0.10	0.09	0.08	0.06	0.26		
HDL cholesterol, mmol/L	−0.08	0.20	−0.07	0.27	−0.14	0.03	−0.09	0.08	−0.05	0.32		
hs-CRP, mg/L	−0.10	0.09	−0.10	0.09	−0.15	0.01	−0.04	0.49	−0.06	0.22		
Urinary creatinine excretion, mmol/24 h	0.24	<0.001	0.25	<0.001	0.25	<0.001	0.07	0.19	0.06	0.22		
Coumarin derivatives, yes vs. no	−0.09	0.13	−0.10	0.11			−0.02	0.67	−0.05	0.34		
Calcineurin inhibitors, yes vs. no	0.15	0.01	0.15	0.02	0.16	0.01	0.20	<0.001	0.16	0.003	0.18	0.001
Sirolimus, yes vs. no	−0.07	0.23	−0.07	0.23			−0.06	0.24	−0.05	0.30		
Insulin therapy, yes vs. no	−0.10	0.10	−0.11	0.08			−0.06	0.26	−0.07	0.19		
Prednisolone, cumulative dose, g ^2^	−0.12	0.05	−0.12	0.05			−0.13	0.01	−0.13	0.01		

^1^ Variables showing *p*-values below 0.10 for the trend of tertiles of IGF1 in at least one of the sexes (see Table 1), with the exception of highly correlated variables (e.g., BMI, waist circumference, serum creatinine), as well as body weight and body height were included for multivariable linear regression analysis. Abbreviations: AST: Aspartate transaminase; eGFR: Estimated glomerular filtration rate; GGT: Gamma-glutamyltransferase; HDL: High-density lipoprotein; hs-CRP: High sensitivity C-reactive protein; SQUASH: Short QUestionnaire to ASsess Health-enhancing physical activity [27]. ^2^ The cumulative dose of prednisolone was calculated as the sum of the maintenance dose of prednisolone until inclusion and the dose of prednisolone or methylprednisolone required for treatment of acute rejection (a conversion factor of 1.25 was used to convert the methylprednisolone dose to its prednisolone dose equivalent).

**Table 3:** Association between log2-transformed plasma IGF1 levels and the risk of all-cause mortality in female and male RTR ^1^.

	277 Female RTR (56 Events)			343 Male RTR (77 Events)		
Variable	HR (log2)	95% CI	*p*-Value	HR (log2)	95% CI	*p*-Value
Crude model	0.42	0.26–0.66	<0.001	0.74	0.52–1.04	0.09
Model 1 ^2^	0.40	0.24–0.65	<0.001	0.85	0.56–1.29	0.44
Model 2 ^3^	0.47	0.27–0.81	0.006	0.88	0.58–1.34	0.55
Model 3 ^4^	0.33	0.16–0.64	0.001	0.88	0.54–1.42	0.60
Model 4 ^5^	0.38	0.23–0.63	<0.001	0.81	0.51–1.27	0.35
Model 5 ^6^	0.39	0.24–0.65	<0.001	0.87	0.57–1.32	0.50
Model 6 ^7^	0.34	0.20–0.57	<0.001	0.94	0.61–1.45	0.78
Model 7 ^8^	0.36	0.21–0.61	<0.001	1.06	0.66–1.69	0.82
Model 8 ^9^	0.41	0.24–0.69	0.001	0.85	0.55–1.29	0.44

^1^ Hazard ratios (HR) per 1 unit increment in log2-transformed plasma IGF1 levels and corresponding 95% confidence intervals (CI) were derived from Cox proportional hazards models. ^2^ Multivariable model adjusted for age and estimated glomerular filtration rate (eGFR). ^3^ Multivariable model adjusted for age, eGFR, body length, body weight, waist circumference, systolic blood pressure, and diastolic blood pressure. ^4^ Multivariable model adjusted for age, eGFR, smoking status, alcohol consumption, and Short QUestionnaire to ASsess Health-enhancing physical activity (SQUASH) score [27]. ^5^ Multivariable model adjusted for age, eGFR, glucose, glycated hemoglobin (HbA1c), triglycerides, serum total cholesterol, high-density lipoprotein (HDL) cholesterol, and low-density lipoprotein (LDL) cholesterol. ^6^ Multivariable model adjusted for age, eGFR, serum creatinine, and urine total protein. ^7^ Multivariable model adjusted for age, eGFR, primary renal disease, graft rejection, dialysis before transplantation, time between transplantation and baseline visit, and donor status. ^8^ Multivariable model adjusted for age, eGFR, aspartate transaminase (AST), gamma-glutamyltransferase (GGT), serum albumin, high sensitivity C-reactive protein (hs-CRP), follicle-stimulating hormone, and luteinizing hormone. ^9^ Multivariable model adjusted for age, eGFR, antidiabetics, antihypertensive drugs, coumarin derivatives, proliferation inhibitors, calcineurin inhibitors, insulin, and prednisolone.

**Table 4:** Mediation analysis of the relationship between plasma IGF1, 24 h urinary creatinine excretion, and all-cause mortality in female RTR.

		Multivariable Model ^1^	
Potential Mediator	Effect ^2^	Coefficient (95% CI, *bc*) ^3^	Proportion Mediated ^4^
24 h urinary creatinine excretion	indirect effect (*ab* path)	−0.11 (−0.18–−0.06)	39.3%
	direct effect (*c’* path)	−0.17 (−0.33–−0.02)	
	total effect (*ab + c’* path)	−0.28 (−0.44–−0.12)	

^1^ Coefficients and corresponding 95% confidence intervals (CI) of the indirect and total effects are standardized for the standard deviations of the potential mediator, plasma IGF1, and all-cause mortality. ^2^ Coefficients are adjusted for age and estimated glomerular filtration rate (eGFR). ^3^ 95% CIs for the indirect and total effects are bias-corrected confidence intervals after running 2000 bootstrap samples. ^4^ The size of (statistically significant) mediated effects is calculated by dividing the standardized indirect effect by the standardized total effect followed by multiplication by 100.

## Supplementary Materials

The following are available online at https://www.mdpi.com/2077-0383/9/2/293/s1. Figure S1: Overview of calibration data for the thirteen runs carried out for quantification of insulin-like growth factor 1 (IGF1) in the clinical samples. Figure S2: Overview of the quality control data obtained during the thirteen runs carried out for quantification of insulin-like growth factor 1 (IGF1) in the clinical samples. Table S1: Association between log2-transformed plasma IGF1 levels and the risk of infectious disease-related mortality in female and male RTR. Table S2: Association between log2-transformed plasma IGF1 levels and the risk of cardiovascular mortality in female and male RTR. Table S3: Association between log2-transformed plasma IGF1 levels and the risk of cancer-related mortality in female and male RTR. Table S4: Association between log2-transformed plasma IGF1 levels and the risk of miscellaneous-cause mortality in female and male RTR. Table S5: Mediation analysis of the relationship between 24 h urinary creatinine excretion, plasma IGF1 levels, and all-cause mortality in female RTR.
