# Supplementary material for: Female Specific Association of Low Insulin-Like Growth Factor 1 (IGF1) Levels with Increased Risk of Premature Mortality in Renal Transplant Recipients
Source: J Clin Med. 2020 Jan 21;9(2):293. doi: 10.3390/jcm9020293 (PMC7073643; doi:10.3390/jcm9020293)
Supplement: Supplementary file 1 [file jcm-09-00293-s001.pdf]

Supplementary Materials

Female Specific Association of Low Insulin-Like Growth Factor 1 (IGF1) Levels with Increased Risk of Premature Mortality in Renal Transplant Recipients

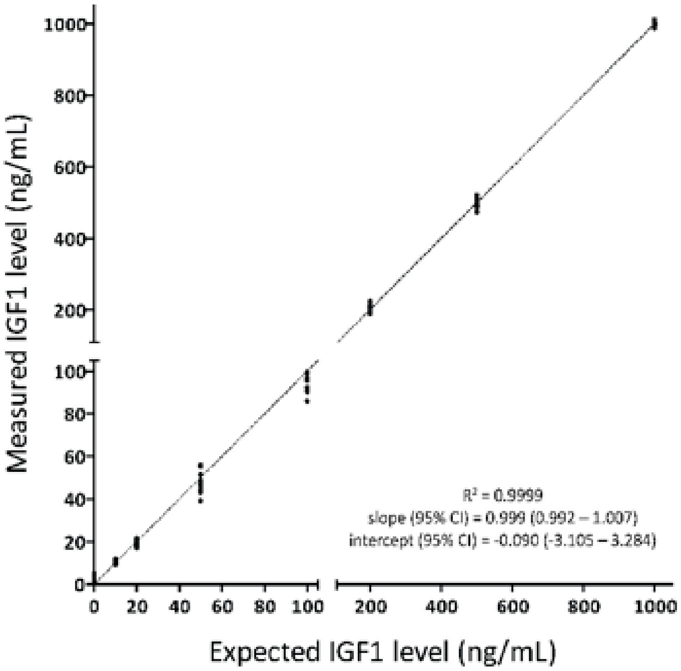

Figure S1. Overview of calibration data for the thirteen runs carried out for quantification of insulin-like growth factor 1 (IGF1) in the clinical samples. The scatter plot features individual data points for all calibrants which were measured during the thirteen analytical runs carried out for clinical sample analysis, and which furthermore met the criteria as stipulated in the United States Food and Drug Administration guidelines on bioanalytical method validation [1]. Coefficients of determination for the thirteen analytical runs were between 0.9977 and 0.9996 based on a 1/x-weighted quadratic regression model. A linear regression curve plus corresponding statistics is included for the linear regression analysis based on the average IGF1 levels of the calibrants.

1. Food and Drug Administration (FDA). Guidance for Industry: Bioanalytical Method Validation; U.S. Department of Health and Human Services: Washington, DC, U.S.A.; 2001.

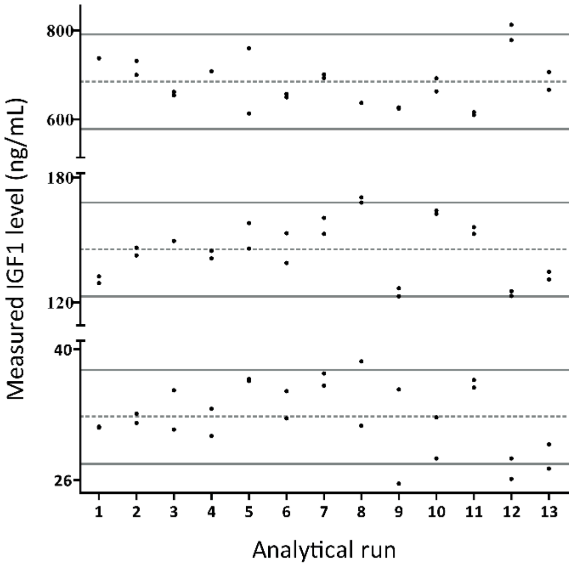

Figure S2. Overview of the quality control data obtained during the thirteen runs carried out for quantification of insulin-like growth factor 1 (IGF1) in the clinical samples. Quality Control (QC) samples with levels around 3-4 times the lower limit of quantification (QC-low), with midrange levels (QC-medium), and with levels around 70% of the upper limit of quantification (QC-high) were processed in duplicate during each analytical run. As is shown in the figure, biases within ± 15% were observed for a sufficient number of QC samples in order to meet the regulatory requirements [1] which specify that at least 4 out of 6 of the QC samples (and at least one of the two samples at the same QC level) should be within ± 15% of their respective nominal value.

1. Food and Drug Administration (FDA). Guidance for Industry: Bioanalytical Method Validation; U.S. Department of Health and Human Services: Washington, DC, U.S.A.; 2001.

Table S1. Association between log2-transformed plasma IGF1 levels and the risk of infectious disease-related mortality in female and male RTR.[1.]

	277 female RTR (16 events)			343 male RTR (21 events)		
**Variable**	**HR (log2)**	**95% CI**	**P value**	**HR (log2)**	**95% CI**	**P value**
**Crude model**	0.18	0.08-0.40	<0.001	0.86	0.43-1.75	0.68
**Model 1** [2]	0.17	0.07-0.38	<0.001	0.84	0.36-1.95	0.68

[1] Hazard ratios (HR) per 1-unit increment in log2-transformed plasma IGF1 levels and corresponding 95% confidence intervals (CI) were derived from Cox proportional hazards models. [2] Multivariable model adjusted for age and estimated glomerular filtration rate (eGFR).

Table S2. Association between log2-transformed plasma IGF1 levels and the risk of cardiovascular mortality in female and male RTR.[1.]

	277 female RTR (19 events)			343 male RTR (32 events)		
**Variable**	**HR (log2)**	**95% CI**	**P value**	**HR (log2)**	**95% CI**	**P value**
**Crude model**	0.45	0.20-1.02	0.06	0.60	0.36-0.99	0.05
**Model 1** [2]	0.43	0.18-1.00	0.05	0.60	0.33-1.09	0.10

[1] Hazard ratios (HR) per 1-unit increment in log2-transformed plasma IGF1 levels and corresponding 95% confidence intervals (CI) were derived from Cox proportional hazards models. [2] Multivariable model adjusted for age and estimated glomerular filtration rate (eGFR).

Table S3. Association between log2-transformed plasma IGF1 levels and the risk of cancer-related mortality in female and male RTR.[1.]

	277 female RTR (12 events)			343 male RTR (12 events)		
**Variable**	**HR (log2)**	**95% CI**	**P value**	**HR (log2)**	**95% CI**	**P value**
**Crude model**	1.14	0.39-3.36	0.81	0.62	0.27-1.41	0.25
**Model 1** [2]	1.50	0.45-4.93	0.51	1.13	0.38-3.37	0.83

[1] Hazard ratios (HR) per 1-unit increment in log2-transformed plasma IGF1 levels and corresponding 95% confidence intervals (CI) were derived from Cox proportional hazards models. [2] Multivariable model adjusted for age and estimated glomerular filtration rate (eGFR).

Table S4. Association between log2-transformed plasma IGF1 levels and the risk of miscellaneous-cause mortality in female and male RTR.[1.]

	277 female RTR (9 events)	343 male RTR (12 events)

Variable	HR (log2)	95% CI	P value	HR (log2)	95% CI	P value
**Crude model**	0.51	0.15-1.68	0.27	1.35	0.50-3.64	0.55
**Model 1** [2]	0.43	0.10-1.78	0.24	2.02	0.63-6.49	0.24

[1] Hazard ratios (HR) per 1-unit increment in log2-transformed plasma IGF1 levels and corresponding 95% confidence intervals (CI) were derived from Cox proportional hazards models. [2] Multivariable model adjusted for age and estimated glomerular filtration rate (eGFR).

Table S5. Mediation analysis of the relationship between 24h urinary creatinine excretion, plasma IGF1 levels, and all-cause mortality in female RTR.

		Multivariable model [1]	
**Potential mediator**	**Effect** [2]	**Coefficient (95% CI, *bc*)** [3]	**Proportion mediated** [4]
**Plasma IGF1 levels**	indirect effect (*ab* path)	-0.05 (-0.11 – -0.01)	9.3%
	direct effect (*c′* path)	-0.45 (-0.60 – -0.29)	
	total effect (*ab* + *c′* path)	-0.50 (-0.64 – -0.35)	

[1] Coefficients and corresponding 95% confidence intervals (CI) of the indirect and total effects are standardized for the standard deviations of the potential mediator, 24h urinary creatinine excretion, and all-cause mortality. [2] Coefficients are adjusted for age and estimated glomerular filtration rate (eGFR). [3] 95% CIs for the indirect and total effects are bias-corrected confidence intervals after running 2,000 bootstrap samples.[4] The size of (statistically significant) mediated effects is calculated by dividing the standardized indirect effect by the standardized total effect followed by multiplication by 100.
